# Wearable adjunct ozone and antibiotic therapy system for treatment of Gram-negative dermal bacterial infection

**DOI:** 10.1038/s41598-022-17495-3

**Published:** 2022-08-17

**Authors:** Alexander Roth, Murali Kannan Maruthamuthu, Sina Nejati, Akshay Krishnakumar, Vidhya Selvamani, Sotoudeh Sedaghat, Juliane Nguyen, Mohamed N. Seleem, Rahim Rahimi

**Affiliations:** 1 Department of Mechanical Engineering, Purdue University, West Lafayette, USA; 2 Birck Nanotechnology Center, West Lafayette, USA; 3 Division of Pharmacoengineering and Molecular Pharmaceutics, Eshelman School of Pharmacy, University of North Carolina at Chapel Hill, Chapel Hill, USA; 4 Department of Materials Engineering, Purdue University, West Lafayette, USA; 5 Department of Electrical and Computer Engineering, Purdue University, West Lafayette, USA; 6 Department of Biomedical Sciences and Pathobiology, Virginia Tech, Blacksburg, USA

**Keywords:** Biomedical engineering, Drug delivery

## Abstract

The problematic combination of a rising prevalence of skin and soft tissue infections and the growing rate of life-threatening antibiotic resistant infections presents an urgent, unmet need for the healthcare industry. These evolutionary resistances originate from mutations in the bacterial cell walls which prevent effective diffusion of antibiotics. Gram-negative bacteria are of special consideration due to the natural resistance to many common antibiotics due to the unique bilayer structure of the cell wall. The system developed here provides one solution to this problem through a wearable therapy that delivers and utilizes gaseous ozone as an adjunct therapy with topical antibiotics through a novel dressing with drug-eluting nanofibers (NFs). This technology drastically increases the sensitivity of Gram-negative bacteria to common antibiotics by using oxidative ozone to bypass resistances created by the bacterial cell wall. To enable simple and effective application of adjunct therapy, ozone delivery and topical antibiotics have been integrated into a single application patch. The drug delivery NFs are generated via electrospinning in a fast-dissolve PVA mat without inducing decreasing gas permeability of the dressing. A systematic study found ozone generation at 4 mg/h provided optimal ozone levels for high antimicrobial performance with minimal cytotoxicity. This ozone treatment was used with adjunct therapy delivered by the system in vitro. Results showed complete eradication of Gram-negative bacteria with ozone and antibiotics typically used only for Gram-positive bacteria, which showed the strength of ozone as an enabling adjunct treatment option to sensitize bacteria strains to otherwise ineffective antibiotics. Furthermore, the treatment is shown through biocompatibility testing to exhibit no cytotoxic effect on human fibroblast cells.

## Introduction

Within the healthcare industry, infections of the skin or other soft tissues are a growing cause of patient morbidity. These skin and soft tissue infections (SSTIs), which often infect pressure ulcers (PUs) or diabetic foot ulcers (DFUs) are part of the large global market for wound care, which is estimated to be $15 billion USD in 2022 and increase to over $22 billion USD by 2024^1^. In the US, SSTIs are the cause for 3.5% of emergency room visits with hospitalization costing patients an average of $8000 USD per stay^2,3^. These numbers are expected to increase even further in years to come due to the prevalence of chronic health conditions such as diabetes, and an aging population. In the US, 34.2 million people (approximately 10% of the population) have diabetes^4,5^. On a global scale, it is estimated that about 2% of adults with diabetes will develop a DFU each year, leading to 9.1 million cases annually, with about half of all DFUs becoming infected^6–9^. Such infections often lead to reduced healing of the wound and other conditions such as osteomyelitis, systemic infection, increased risk of amputation, and death^10–13^.

Typical treatment for SSTI infections, including those in PUs DFUs, involves administration of antibiotics. While this treatment method is able to reduce bacterial load in many cases, it does nothing to help promote early wound healing. Additionally, bacterial resistance to antibiotics is a growing global issue that further reduces the validity of current treatment methods^14–16^. Gram negative (G − ve) bacteria exhibit a natural resistance to many antibiotic treatments due to an additional outer membrane in the cell structure which prevents many antibiotics from reaching their intended target within the cell and can be altered more easily to develop new resistances^17,18^. This has led to such an increase in number and severity of multidrug-resistant G − ve bacteria that the World Health Organization (WHO) included only G − ve bacteria in their list of antibiotic resistant strains requiring the most urgent soutions^18^. This issue is even more alarming because the development and approval of new antimicrobials effective in treating multidrug-resistant G − ve pathogens has not kept pace with the continued emergence of new resistances in bacteria. This is due to the long, expensive, and high-risk process of drug development that disincentivizes production and undergoing the arduous task of regulatory approval for the marketplace^19^. As a result, there is an urgent need for the development of alternative treatment options for SSTIs.

This great need has led to investigation into a number of alternative therapies that could be used against infections caused by virulent G − ve bacteria strains. Among the most popular are the use of cold atmospheric plasma (CAP), metallic nanoparticles (NPs), and gaseous ozone. Previous research using CAP has shown that the ionized particles generated exhibit encouraging antimicrobial properties and also help promote healing factors in the wound. Unfortunately, these systems requires specialty equipment and trained personnel to function, which prohibits utilization of the technology for frequent treatments^20,21^. Metallic NPs, such as those made from copper and silver, have also been extensively studied because of their strong antimicrobial properties. Although nanoparticle-based metals have found a wide range of applications in topical wound therapy, their main drawback is high levels of toxicity to natural tissue^22–24^. Even more alarming are the reports of some G − ve bacteria strains forming resistances to silver^25^. Additionally, antimicrobial patches are being developed with novel materials such as chitosan for microneedle patches and nanofiber mats. These patches are used as delivery methods for wound healing factors and antimicrobial substances to promote wound health as well^26–29^. While such systems and platforms have provided new routes for more effective and deeper delivery of therapeutics, they often have a limited amount of drug during which makes them impractical for frequent applications with chronic wounds^30^. Additionally, these systems still utilize common antibiotics and nanoparticles, which persist in their limited applications due to bacterial resistance and cytotoxicity respectively. Gaseous ozone, on the other hand, has been shown to be a strong, safe, and accessible alternative treatment for many years. Studies have shown that topically applied gaseous ozone is effective in eliminating a wide range of harmful microorganisms including bacteria, viruses, fungi, and more^31^. This is due to its naturally strong oxidative tendencies which work to weaken the outer membrane of the bacteria cell through applied oxidative stress^31^. The historic success of ozone as an antimicrobial has led to a wide range of studies for clinical applications. Many of the in vitro tests have focused on utilization of high concentrations of ozone (0.6–20 μg/mL). The results indicate that at such levels, ozone is able to eliminate bacteria over very short exposure times^32,33^. An additional benefit of ozone that has been studied is its ability to stimulate early wound healing activity in cells, which is also tied to the application of oxidative stress^34–39^. Although high concentration treatments are able to treat infections more quickly, they also require special equipment and facilities and can damage healthy tissue through over-exposure. However more reason advancements of electronic systems and the power photosystems have provided the capability to generate ozone relatively lower concentration through miniaturized corona discharge systems. Such systems can provide the unique possibility of providing more sustained and low levels of ozone to the targeted wound site with a portable generation approach, which eliminates the need of specialized equipment and trained personnel and a greatly reduced risk of damaging healthy cells^32,40^.

In addition to using ozone therapy as a stand-alone treatment, it is proposed that combining ozone as an adjunct therapy with current antibiotics would improve the performance of both therapies significantly, especially against resistive strains of G − ve bacteria. Using an adjunct therapy to increase antibiotic efficacy has been studied previously with electroporation, chemical photosynthesis of reactive oxygen species (ROS), and intraperitoneal injection of ozone^41–43^. The enhanced efficacy of antibiotics in such combination therapy systems has been explained by the process of increased diffusion of antibiotics into the bacteria cells through the secondary treatment which damage the outer membrane of the bacterial cells. While these methods have shown positive initial results, there are significant limitations of each including limited penetration depth due to cytotoxicity of electroporation, chemical synthesis needed for photogeneration of ROS, and the invasive procedure for ozone injection. Topical ozone applied through a wearable patch can provide the same synergistic properties by oxidizing the cell membrane and creates holes for the antibiotic to pass through into the cell^44^. This technique is effective in generating a synergistic effect between the adjunct and antibiotic therapies. One additional benefit of such treatments is that the compromised bacterial membranes will increase the number of available antibiotic treatment options. Because of the reduction of the outer membrane defenses, which are the main differentiation between Gram-positive (G + ve) and G − ve strains, it is expected that the adjunct ozone therapy will enable antibiotics that are commonly effective on G + ve to effectively work on G − ve strains of bacteria. Using ozone to bypass intrinsic or developed antibiotic resistances of G − ve bacteria will enable the prolonged use of current antibiotic technologies. The two combined therapies will also enable a reduced dosage of both antibiotics and ozone, limiting the negative health effects of high-concentration ozone exposure and slow the rate of new antibiotic resistances being developed.

**Figure 1:**
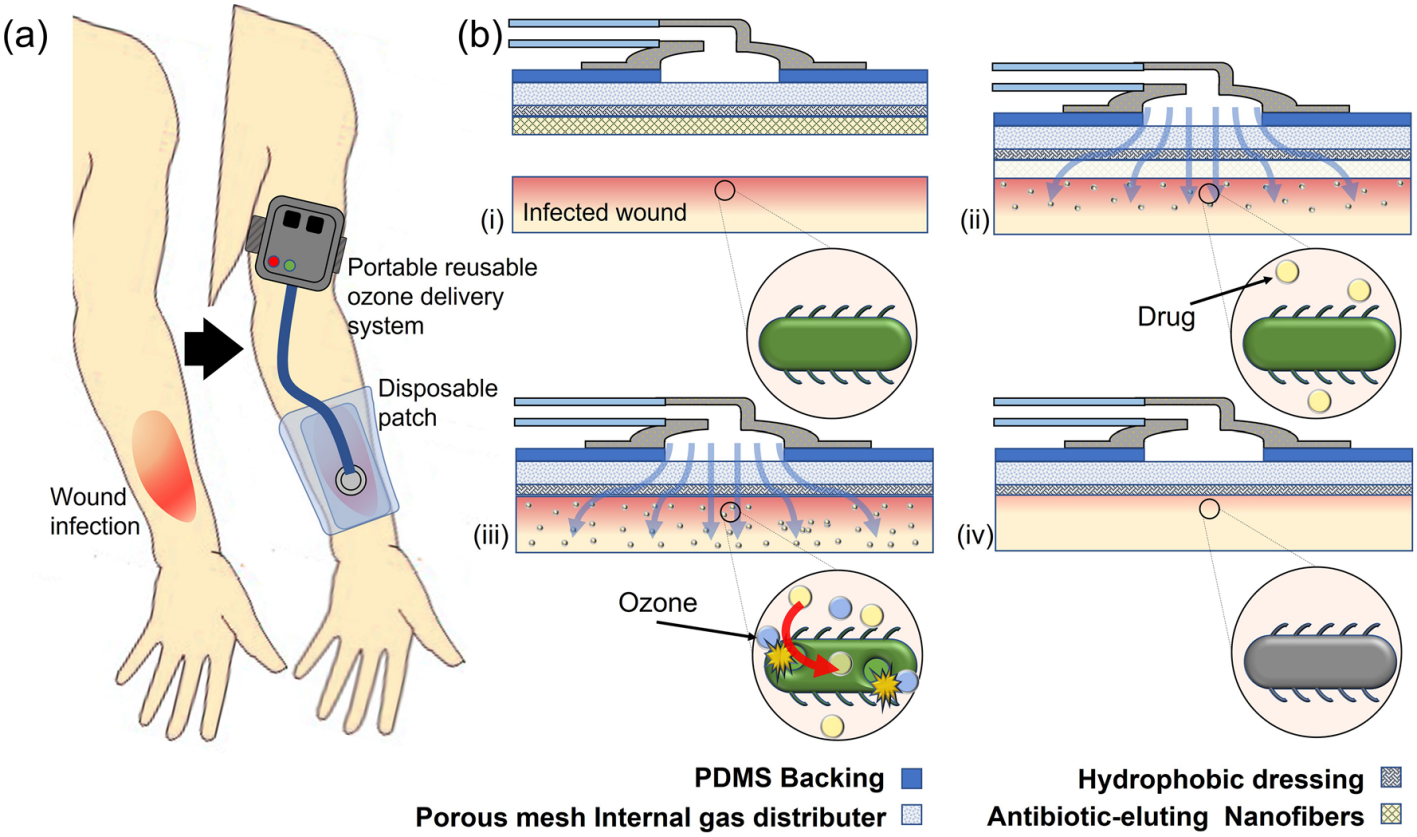
Wearable adjunct ozone and topical antibiotic therapy system. (**a**) Ozone and antibiotic adjunct therapy can be used as an alternative treatment for skin and soft tissue infections that do not respond to traditional therapies. Ozone provides antimicrobial properties and enables antibiotic to enter cell and disrupt cell functions, such as protein production. (**b**)The system utilizes gaseous ozone and gas permeable and drug-eluting nanofiber mat for treating developing wounds in the following process: (i) All-in-one wound patch with drug-eluting nanofiber mesh and gas permeable membrane is applied to skin wound. (ii) NFs begin to dissolve and release the topical antibiotics. (iii) Ozone is applied to the system for full treatment duration as topical antibiotics are completely released from NFs. Ozone and antibiotics work together to eliminate infection. (iv) Once the wound has healed, the wound patch is removed from the area. Combination of ozone and antibiotic treatment can treat antibiotic resistance infections and prevent development of new infections, leading to faster healing times.

Here, we describe the development of a novel integrated therapeutic system for administering topical adjunct ozone and antibiotic treatment to infected dermal wounds, especially those caused by drug resistant G − ve bacteria that require novel antimicrobials. This system is comprised of two parts, a portable ozone generation unit and a disposable application patch for interfacing with the wound surface. The ozone generation system contains a low-power ozone generator and microblower that are controlled by an onboard battery pack and microcontroller, enabling controlled delivery of ozone from 0 to 4 mg/h. The wound patch features a three-layer structure containing an internal diffusion layer to enable more uniform ozone application by causing physical spreading of the flow through the pores, a hydrophobic and gas-permeable membrane for prevention of fluid uptake into the dressing, and a biodegradable nanofiber (NF) drug-eluting mesh for topical application of antibiotic^45^. This will create a fully integrated system to enable topical adjunct therapy of antibiotics with gaseous ozone. The patch is applied directly to the wound site. There, the bio-dissolvable polymer fibers being to break down upon contact with the wound bed and release the antibiotic payload topically to the wound. Concurrently, gaseous ozone is pumped from a portable external ozone generation system through the attached patch and onto the surface of the wound as shown in Fig. 1, providing additional antimicrobial action and increasing the antibiotic effect via increased diffusion due to oxidative portion of the bacterial membranes.

For the system to be effective, the following engineering criteria were considered. First, the portability of the system is important to ensure patients were able to access the treatment for extended periods of time without being confined by clinical equipment or immovable tethers, so that patients can easily self-administer their medication at home descretely and without interfering with their lifestyle. The portable power source is capable of powering a single generator, producing 0–4 mg/h, or two generators producing 0–8 mg/h of ozone. To identify the optimal ozone generation rate, a systematic investigation of antimicrobial and biocompatible properties of ozone at varying generation rates was conducted. Second, the optimized ozone system was tested in combination with topical antibiotics to study the synergistic enhancement of treatment of G − ve bacteria in physiologically relevant conditions. These treatments focused on utilizing ozone to sensitize G − ve bacteria to antibiotics commonly used on G + ve pathogens. Because the application of the gaseous ozone and the antibiotic must be able to exist concurrently, typical topical administration of antibiotics, such as a cream, creates a barrier to diffusion of the ozone. As such, the reported system uses a mat of bio-dissolvable nanofibers (NFs) to topically delivery the antibiotics. In this study, we selected two common antibiotics for treating G + ve infections, vancomycin and linezolid, to be used as a proof of concept. The combination of ozone and these antibiotics was shown in the study to cause to a significantly increased treatment efficacy. Therefore, there is reason to believe that ozone is a key adjunct treatment for giving previously resisted antibiotics new life. In addition, the system was designed for the translation of the product through clinical trials to the marketplace by using low-cost materials (disposable patch cost: < $2.50) and a topical treatment method that would be easy to implement in the clinical setting^37^.

## Results and discussion

### Portable ozone delivery system

**Figure 2:**
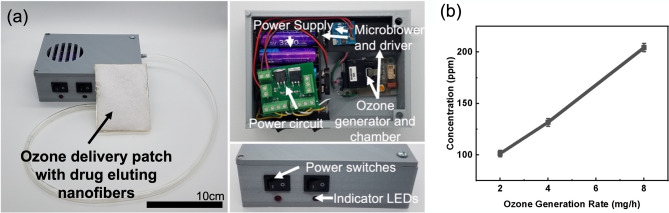
Portable adjunct ozone and topical antibiotic therapy system. (**a**) Ozone wound treatment system designed to administer adjunct ozone and antibiotic therapy topically to dermal wounds. System comprised of portable ozone generation system with microblower for ozone delivery and a porous mesh of drug-eluting PVA nanofibers for antibiotic delivery. Portable rechargeable system is fitted to custom housing and utilizes onboard low-power electronics for. (**b**) Relationship of concentration of ozone created by portable system to the mass generation rate. Error bars denote standard deviation.

An ozone generation and delivery system was designed to enable the topical ozone and antibiotic wound therapy (Fig. 2a). The system features an onboard ozone generation system that uses corona discharge to produce gaseous ozone from ambient air and a microblower system to deliver the generated ozone. An onboard battery powered the system with switches for user control of both ozone and blower function. The design enables point-of-care generation and application of ozone from a wearable and reusable system for easy incorporation into current clinical practices. Ozone generation levels of the system were characterized to quantify the output of ozone at each generation setting. The output of the ozone generator was controlled through a microcontroller pulse width modulation (PWM) signal and the concentration of ozone in the flow output was measured using a commercial ozone sensor. Figure 2b shows the results of the concentration of ozone measured in triplicate at three different mass generation settings on the developed portable ozone delivery system. Ozone levels were measured at 100 parts per million (ppm) for 2 mg/h, 132 ppm for 4 mg/h, and 204 ppm for 8 mg/h generation rates. This shows that the ozone concentration in ppm is roughly linear in response to the mass generation rate of the system when a baseline generation offset of ozone is considered. This controllable behavior enables investigation of optimized generation rates for the application ozone treatment.

### Antibiotic eluted nanofibers characterization

The adjunct therapy patch designed for this system is comprised of three layers, an internal dispersion layer to increase the coverage area of the ozone, a hydrophobic dressing for interfacing with the wound, and a biodegradable drug-eluting NF mesh. The internal dispersion layer was created from a porous polymer mesh with pore sizes from 0.003 to 0.02 mm^2^ (measured with ImageJ from microscope images) to enable to ozone gas to diffuse throughout before crossing the permeable dressing into the wound bed. This was shown previously to significantly increase the area of and uniformity of the ozone coverage on the woundsite^37^. The gas permeable membrane was treated with polydimethlsiloxane (PDMS) to induce hydrophobicity. This treatment prevents fluid uptake into the dressing pores, which would negatively affect the ozone diffusion into the wound bed for treatment^37^. Because traditional methods of topical antibiotic application, such as creams, inhibit ozone diffusion, the biodegradable NF mesh was used. This mesh was created from poly(vinyl alcohol) (PVA) and antibiotic solution using an electrospinning process. This enabled creation of nano-sized polymer strands to be created in a gas-permeable overlapping mesh structure directly onto the application patch.

**Figure 3:**
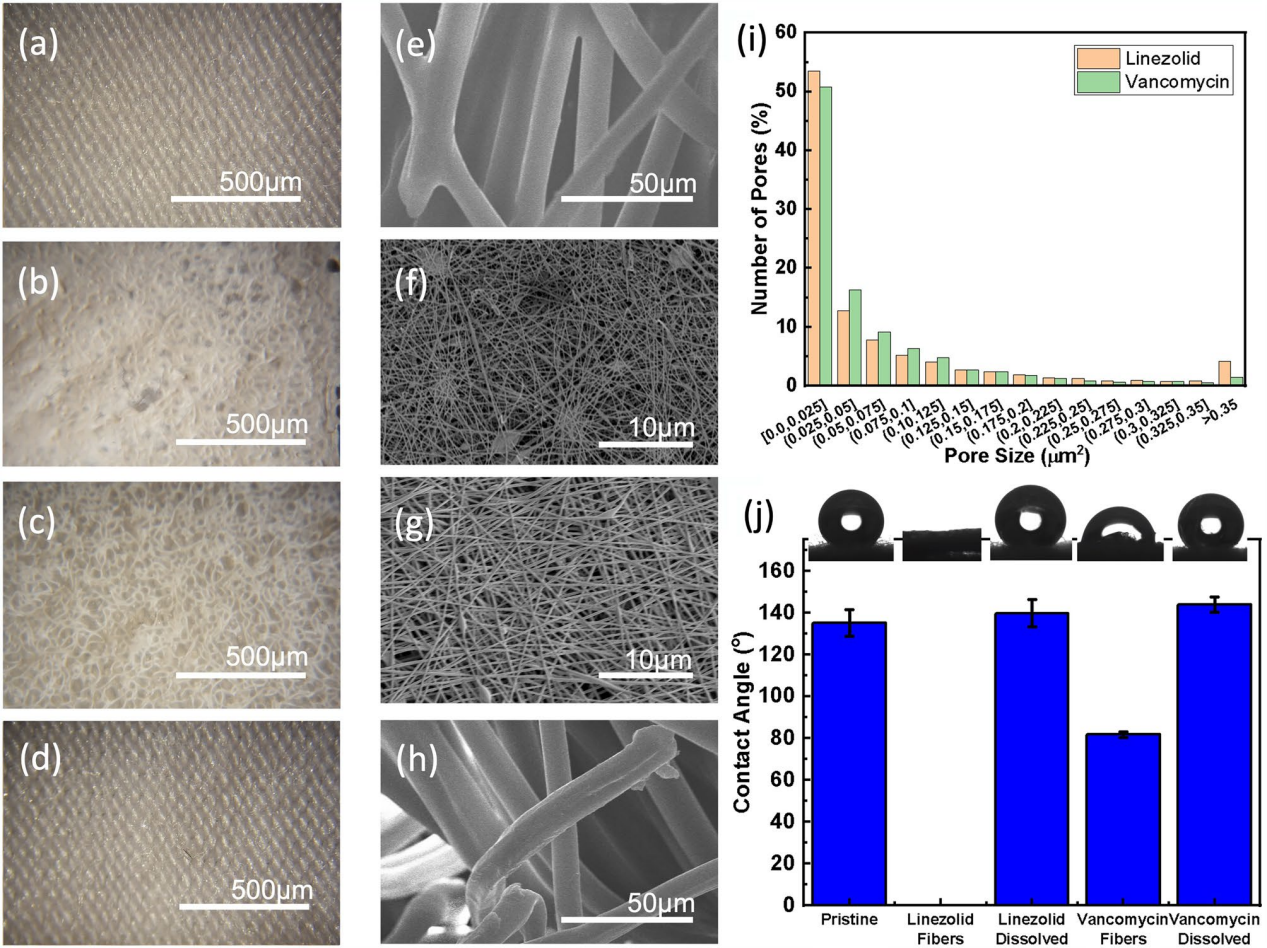
Properties of electrospun NF mat. Microscope image of (**a**,**e**) ozone delivery patch surface and (**b**,**f**) after coating with linezolid containing NFs, (**c**,**g**) and after coating with vancomycin containing NFs, (**d**,**h**) after the dissolution of the NFs. Images were taken using optical microscope (**a**–**d**) and SEM (**e**–**h**). (**i**) Histogram displaying frequency of pore size within vancomycin and linezolid spun fiber mats. (**j**) Contact angle measurement of dressing at various stages of treatment. Error bars denote standard deviation.

In order to identify the structure and properties of the NFs, microscope and Scanning Electron Microscope (SEM) imaging were performed on the NFs that were generated through the electrospinning process. Figure 3a–h shows the imaging results from both an optical microscope (OM) and the SEM. Image comparison of the PDMS treated dressing before and after NF application and dissolution shows that there is no change to the structure caused by deposition and dissolution of the drug eluted NFs. This property was further confirmed and quantified in characterizations below. Viewing the images of the NFs deposited on the dressing, it can be concluded that the mesh generated is again porous in structure. The fiber size was measured to be 300 nm diameter for the fibers containing vancomycin and 100 nm diameter for the fibers containing linezolid. This difference in size is expected to be caused by the larger molecular size of vancomycin (1449.3 Da compared to 337.3 Da for linezolid)^46,47^.

Further analysis was performed with the ImageJ software (Fig. 3i). The size of pores within the electrospun mesh created with both antibiotics was measured. The data was compiled into a histogram. Results indicate that there is little variation between the pore sizes in each mesh, with both having a majority of pores (50.8% for vancomycin and 53.5% for linezolid) under 0.025 μm^2^, though the linezolid mesh did have a larger proportion of pores greater than 0.35 µm^2^ (4.1% vs. 1.4%). This indicates that the porosity due to fiber conglomeration should be similar between the two meshes, with any variation coming from the increased mass of linezolid fibers deposited.

The hydrophobicity/hydrophilicity of the porous dressing was also important for topical application of ozone. High levels hydrophilicity of the NF layer will accelerate fluid interaction and lead to fast dissolution and antibiotic application. On the other hand, high levels of hydrophobicity are necessary for the wound dressing underneath in order to repel fluid uptake into the dressing pores that would inhibit ozone movement from the patch into the wound site. Due to this, the hydrophobic nature of the dressing was investigated. The contact angle was measured on dressing samples before NF deposition, on dressing samples with NF coating, and on dressing samples after NFs were completely dissolved off the surface. The contact angle of the sample corresponds to the hydrophobicity. As described in our previous work, hydrophobic properties were instilled into the dressing through a diluted PDMS coating on the fibers^37^. It was observed that the contact angle of the PDMS treated dressing was 135° before NF deposition and 140+° after the NF had been fully dissolved from the surface. It can also be seen that both the NFs with linezolid and vancomycin have much lower contact angles, 0° and 82°, respectively. These results indicate that the hydrophobic behavior of the engineered dressing does not change after the NFs have been dissolved from the patch surface (Fig. 3j). Thus, the desired hydrophobicity of the patch is maintained throughout the course of the treatment time, while the hydrophilic nature displayed by the NF layer enables rapid dissolution through increased fluid contact.

### Porosity and dissolution

Another property important to the overall performance of the treatment is the porosity of the dressing with and without the NF layer, as the porosity of the dressing allows the gaseous ozone to permeate and topically affect the wound area. The effect of mesh porosity was quantified by measuring the internal flow pressure as a constant flow rate was pumped through the dressing. The state of the dressing was varied to characterize this at different times in the treatment process.

**Figure 4:**
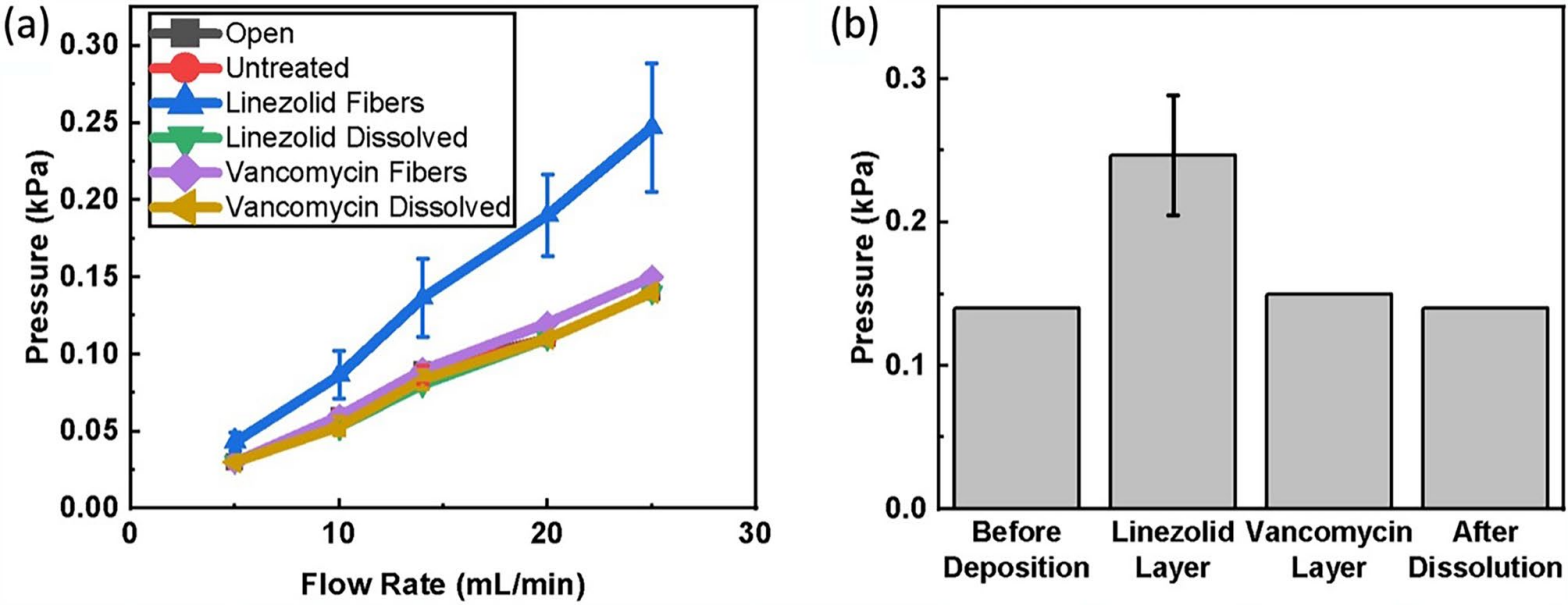
Porosity characterization of ozone dressing with and without drug eluting NFs coating. (**a**) Internal flow resistance at varying flowrates for dressing at different stages of application. (**b**) Comparison of internal flow resistance at 25 mL/min. Error bars denote standard deviation.

Figure 4a shows the measured internal pressure as air was pushed through at flow rates ranging from 5 to 25 mL/min. Samples tested include a baseline flow through the system with no patch (open), a pristine sample of the hydrophobic dressing (untreated), and hydrophobic dressing samples with NFs and after NF dissolution. It can be seen that there is no discernable increase in the resistance to flow for any of the samples except for the dressing with deposited linezolid fibers. The resistance to flow increased by about 80% at the highest flow rate (Fig. 4b). This increase was due to the addition of a significant layer of drug-eluting NFs. NFs were deposited such that the layer on the patch contained antibiotics to the inhibitory concentration of 20 μg/cm^2^. Differing solubilities of vancomycin and linezolid led to a thicker layer of linezolid fibers than vancomycin, as more vancomycin was loaded into each fiber per unit mass (0.1% w/w vancomycin versus 0.03% w/w linezolid). Because many more linezolid fibers are deposited than vancomycin fibers (667 μg/cm^2^ vs 100 μg/cm^2^ for vancomycin), there was a much larger effect on the resistance to gaseous flow despite the relative similarity in pore size discussed in the previous section. Still, even the increased levels of porosity did not prohibit gaseous flow, as the overall effect was reduced due to the porous mesh structure of the NFs seen in Fig. 3. Additionally, the fast-dissolving nature of the fibers (as discussed below) means this temporary decrease in porosity is expected to be negligible.

There is extensive research that validates the use of NFs in drug delivery with a wide range of designed release times from minutes to days^48–56^. Characterization of the dissolution time of the NFs enables insight into the rate at which the active antibiotic is applied to the wound area for treatment and the duration during which the porosity of the dressing is reduced in the case of linezolid NFs. It is beneficial for the antibiotic to be released quickly in this case to enable interaction with bacterial cells as molecular uptake is increased due to the pores in the bacterial cell membranes caused by ozone^57,58^. To characterize the dissolution time, NFs containing a dye with a molecular weight similar to each antibiotic were electrospun under the same conditions as models for the dissolution of the drug-eluting NFs. Each NF sample was cut to size and exposed to DI water for a designated duration. Optical absorbance measurements were taken from the fluid samples and compared to a fully dissolved sample reading. This data was then organized to show the dissolution percentage over time.

**Figure 5:**
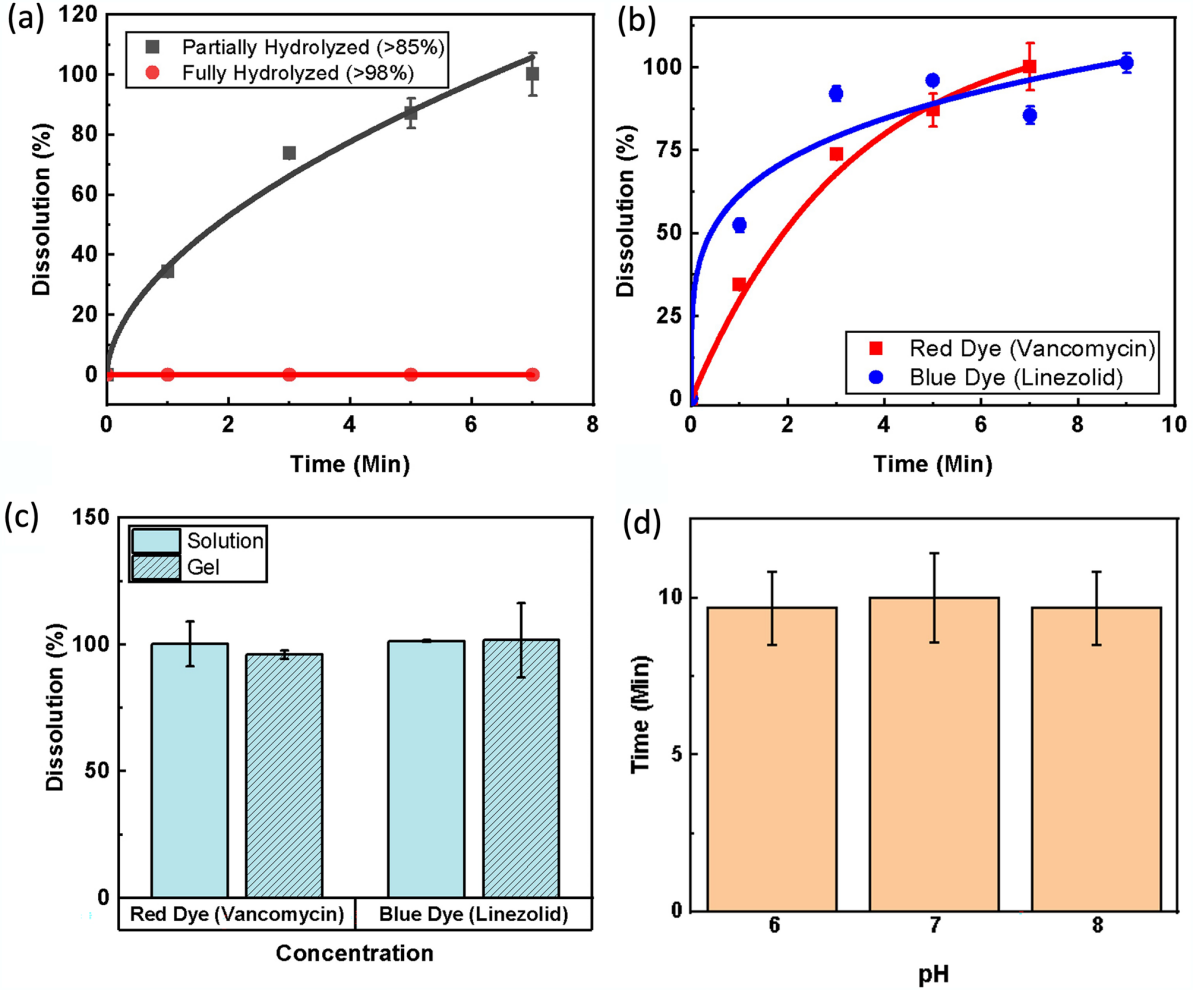
Dissolution characterization of drug eluting NFs. (**a**) Dissolution rate of NF fabricated with partially hydrolyzed and fully hydrolyzed PVA. (**b**) Dissolution over time of partially hydrolyzed NFs infused with drug mimicking dies (Red for vancomycin and Blue for linezolid). (**c**) Proportion of material dissolved by critical time of 10 min (< 3% total treatment duration) for NFs in liquid and gel media. (**d**) Comparison of time needed to achieve critical dissolution of blue dye (linezolid) NFs in buffer solution with different pH levels. Error bars denote standard deviation.

Before fully characterizing the dissolution rate, a study was performed to understand how the hydrolyzation of the PVA used to generate the fibers affects the solubility. Hydrolyzation is a property of PVA that indicates the degree to which the acetate groups are removed during the synthesis process from polyvinyl acetate^59,60^. The effect of hydrolyzation was tested as seen in Fig. 5a. Over time, fully hydrolyzed NFs did not dissolve, while partially hydrolyzed fibers dissolved quickly. This is due to strong hydrogen bond interactions that keep fully hydrolyzed fibers from dissolving below 80 °C. Thus, the partially hydrolyzed PVA was chosen as optimal for fast-dissolving fibers. The dissolution of the NFs was then fully characterized for both model linezolid and vancomycin NFs. As indicated in Fig. 5b, both fibers showed a high dissolution rate. The model linezolid fibers were seen to start dissolving faster, likely due to the smaller size, but the model vancomycin fibers reached full dissolution first (7 min) due to the smaller mass of fibers that needed to be dissolved. Still, the linezolid fibers reached full dissolution after 9 min. Based on this observation, the patch will require application onto the wound for a few minutes before activating the ozone delivery to ensure full dissolution of fibers and diffusion into the wound site before ozone is applied.

It is important that the NFs used for the application of the antibiotics can dissolve in a short duration when compared to the total treatment length to maximize the duration that both components of the adjunct therapy are active within the wound bed. It was determined that a total dissolution time of up to 10 min should be more than sufficient to allow for proper activity of both the ozone and antibiotics used in treatment. NFs were tested to meet this criterion in DI water (as previously described) and in agar gel to mimic a simulated semi-solid wound environment. Figure 5c displays the results, indicating that even in the simulated wound gel environment, the NFs were fully dissolved within the desired 10-min timeframe. Lastly, because the pH found in an infected wound bed can vary, the dissolution of the model linezolid fibers in solutions was characterized with three different pH values. The fibers were again cut to size and exposed to buffer solutions with pH values of 6, 7, and 8. Figure 5d shows a comparison of time needed to reach critical dissolution (> 80%) in each pH. It can be seen from the results that the pH of the solution had very little effect on dissolution time.

### Antibacterial and cytotoxicity optimization of gaseous ozone application

**Figure 6:**
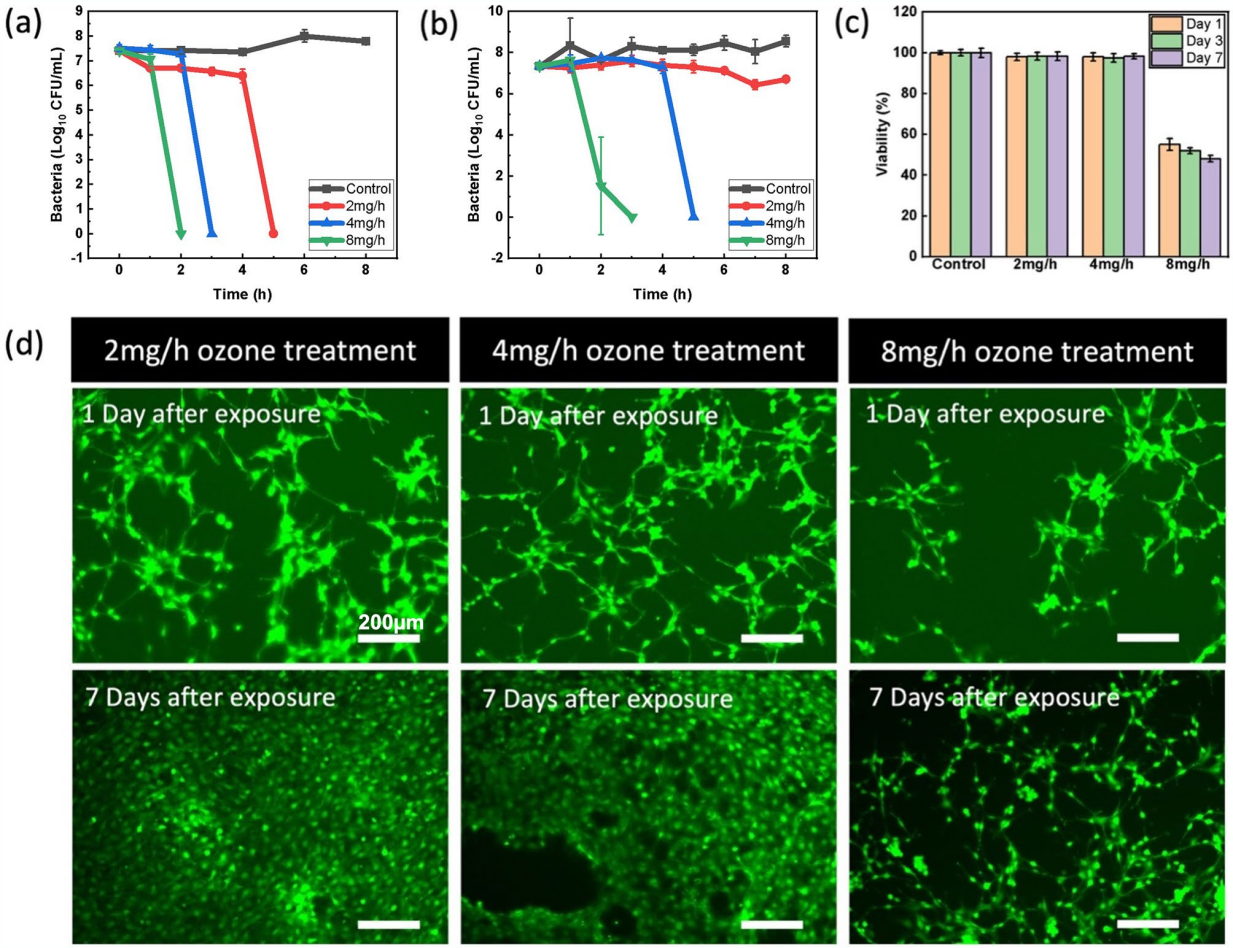
Antimicrobial efficacy and cell viability under continuous exposure to differing levels of ozone (2–8 mg/h). Antimicrobial effect against (**a**) *P. aeruginosa* and (**b**) *E. coli* bacteria cultures in PBS over the course of 8 h. (**c**) Cell viability of human fibroblasts treated with 6 h of ozone at 2–8 mg/h at 37 °C. Viability was measured 1 day, 3 days, and 7 days after treatment ended. (**d**) Live/Dead staining of human fibroblast cells exposed to varying levels of ozone therapy 1 day and 7 days after treatment. Error bars denote standard deviation.

Before assessing the combination therapy, a systematic study of ozone treatment levels was conducted to identify the appropriate concentration of ozone therapy to maximize bactericidal properties while having minimal toxic effect on human cells. For this test, three different ozone generation rates, 2 mg/h, 4 mg/h, and 8 mg/h, were tested as treatment of *P. aeruginosa* and *E. coli*, two of the most common G − ve bacteria in wound infections^61^. Bacteria cultures were suspended in PBS for best observation of antimicrobial properties of each generation rate. PBS was chosen as the test culture medium for these experiments to provide a homeostatic environment where the bacteria were neither actively dying nor reproducing. This allowed for explicit comparison of antimicrobial properties of the ozone treatment at different application levels. Figure 6 shows that in both strains, higher ozone generation rates produced faster kill-off as expected, while *P. aeruginosa* showed higher sensitivity to ozone treatment overall. *P. aeruginosa* cultures were cleared from test wells after 5, 3, and 2 h respectively for 2 mg/h, 4 mg/h, and 8 mg/h ozone generation rates, showing a more significant increase in efficacy when increasing generation from 2 to 4 mg/h than when further increasing from 4 to 8 mg/h. The results on *E. coli* support this trend as well. Total kill off of *E. coli* cultures was not observed in 8 h at ozone generation rate 2 mg/h, while 4 mg/h showed clearing after 5 h and 8 mg/h after 3 h.

The effect of varying ozone generation rates of human fibroblasts was also investigated. Figure 6c shows the percentage of healthy cells after 6 h of ozone exposure at each generation rate 1 day, 3 days, and 7 days after exposure which can be seen imaged in Fig. 6d. It can be seen that higher levels of ozone, namely 8 mg/h, induce stress on cells, leading to a 50% reduction in viability, while both 2 mg/h and 4 mg/h showed no signs of toxicity. Considering both the antimicrobial performance and cytotoxicity of each condition, 4 mg/h showed capability to provide fast and effective antimicrobial properties with minimal cytotoxicity, and was therefore chosen as the appropriate ozone generation setting.

### Adjunct antibacterial therapy

Because the wound environment is conducive to bacterial growth, the accelerated rate of replication will necessitate a higher concentration of ozone for sufficient antimicrobial properties. While effective, this can result in cytotoxic side effects for the human cells. To counteract this concern, a systematic study of the combination effect of antibiotics with adjunct ozone therapy is presented to provide increased antimicrobial action without harmful side effects. To show the positive impact of using the adjunct therapy, the ozone and antibiotic treatment was tested on strains of *P. aeruginosa* and *E. coli,* G − ve bacteria commonly found in SSTIs. For these tests, two antibiotics commonly used to treat G + ve bacteria, vancomycin and linezolid, were selected. By selecting antibiotics typically used only on G + ve bacteria, these tests were able to demonstrate proof of concept for ozone as an adjunct therapy to sensitize G − ve bacteria to resisted antibiotics. Additionally, treatment was conducted on bacteria cultures in tryptic soy broth growth media and 37 °C. These conditions, optimal for bacteria growth, were chosen to show the strength of the adjunct treatment in an environment that matches or exceeds those of a natural wound for promoting bacteria growth. Additionally, the growth of bacteria, which is present in both an infected wound and the simulated conditions presented here is necessary for viewing the full effect that the antibiotics have on the bacteria.

**Figure 7:**
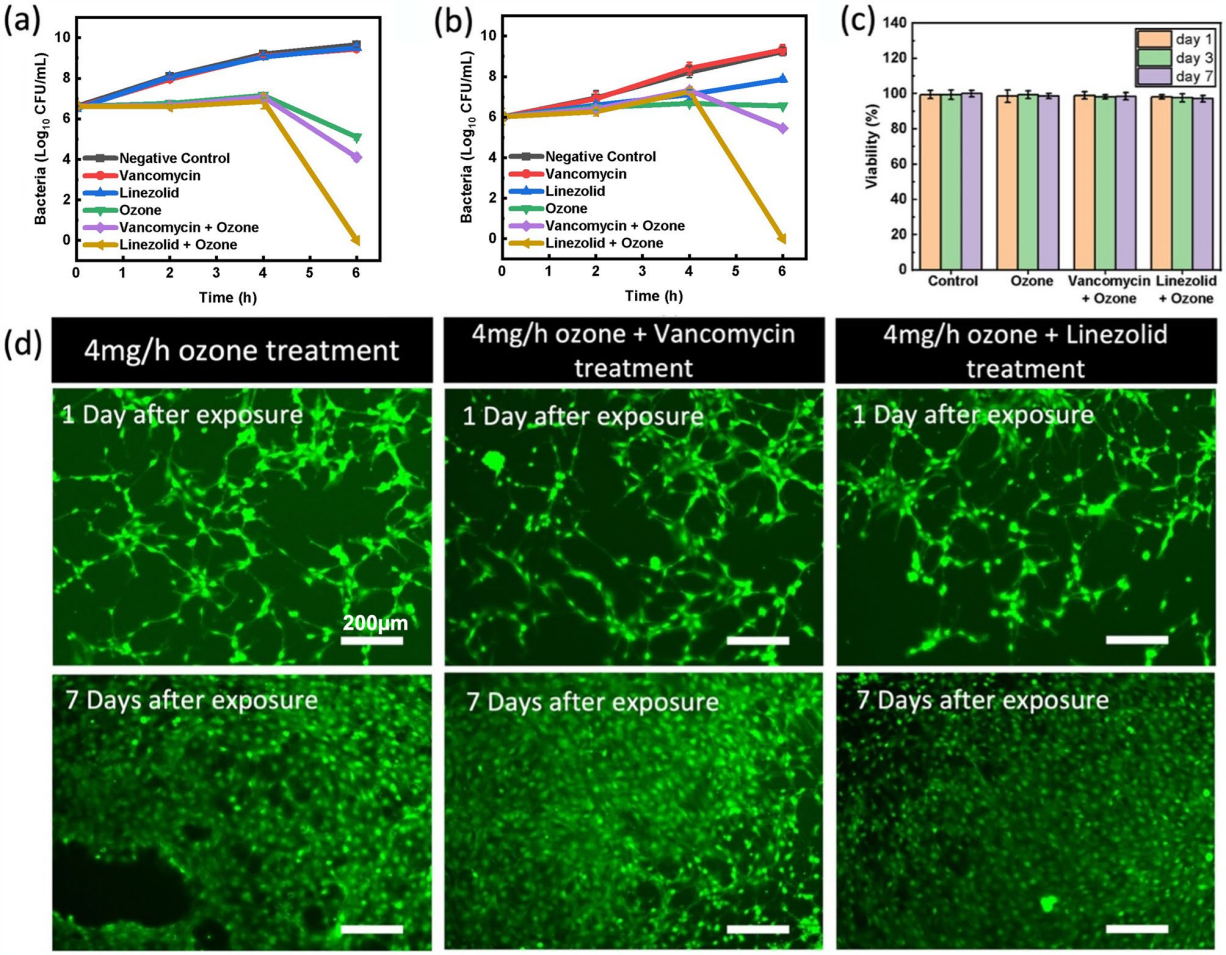
Antibacterial efficacy against bacteria cultures in TSB media and cell viability results of adjunct ozone and antibiotic therapy test in vitro at 37 °C. (**a**) Results for ozone + linezolid and ozone + vancomycin adjunct therapy on *P. aeruginosa*. (**b**) Antibacterial results of ozone + linezolid and ozone + vancomycin adjunct therapy on *E. coli.* Ozone was applied at 4 mg/h for 6 h. Linezolid and vancomycin were applied in solution at 200 μg/mL. (**c**) Viability of human fibroblast cells exposed to 6 h of ozone, ozone + vancomycin, and ozone + linezolid treatment measured 1 day, 3 days and 7 days after treatment ended. (**d**) Live/Dead staining of human fibroblasts 1 day and 7 days after treatment ended. Error bars denote standard deviation.

Figure 7 shows the results of the antibacterial properties and biocompatibility results of the adjunct therapy. Figure 7a indicates that the adjunct therapy of linezolid and gaseous ozone showed a significant increase in antibacterial activity. When compared to the initial bacterial CFU/mL measurement, both the negative control (no treatment) and linezolid and vancomycin controls (antibiotics only) showed significant growth in population as the healthy bacteria in media continued to proliferated. This shows that the antibiotics on their own did not inhibit the growth of the bacteria at all as expected. The ozone sample, which was exposed to 4 mg/h ozone for 6 h, showed a modest bacterial reduction of 1.52 log_10_ CFU/mL. When combined with vancomycin, adjunct treatment showed a significant reduction of 2.52 log_10_ CFU/mL reduction. Additionally, ozone combined with linezolid showed even greater efficacy with complete elimination of all bacteria (6.62 log_10_ CFU/mL). Statistical analysis indicated statistically significant outcomes for both ozone and combination therapies (p < 0.0001). In both cases, the results indicate that the ozone and antibiotic treatments together are more effective than the sum of the parts.

The same test procedure was also performed on *E. coli*, another G-ve bacteria commonly found in dermal wounds. Figure 7b shows the results for both the vancomycin and linezolid adjunct therapies. As seen with *P. aeruginosa*, the negative control and antibiotic controls showed no inhibition of bacteria growth over the 6-h treatment. The ozone therapy alone showed no decrease in the bacteria population, while the combination therapy again showed a significant increase in efficacy, with linezolid adjunct therapy completely eliminating the bacteria (6.02 log_10_ CFU/mL reduction) and vancomycin adjunct therapy enabling a 0.57 log_10_ CFU/mL reduction. The difference in performance of linezolid and vancomycin can be explained by the two different molecules and mechanisms of action. Vancomycin is known to function by binding to peptide layer of the cell wall, which inhibits crosslinking and leads to cell lysis during division. Because vancomycin is a large molecule (1449.3 Da), it is generally prohibited from accessing the peptide layer in G − ve bacteria due to the additional outer lipid layer present is such strains^62,63^. Ozone is hypothesized to enable some vancomycin molecules to bypass this protective layer via oxidative disruption, but the efficiency is not enough for full strength action. Linezolid, on the other hand, is a much smaller molecule (337.3 Da) which inhibits bacteria reproduction by binding to RNA strands necessary for protein production^64,65^. Because of its smaller size, linezolid is more easily able to access the bacteria cell through the oxidative holes that are created by the ozone. Additionally, the combination of two different mechanisms of action applied by ozone and linezolid is able to show a greater efficiency at killing off bacteria under the same application conditions. These results confirm the benefits of using gaseous ozone as an adjunct therapy to sensitize G − ve bacteria to antibiotics typically used only for G + ve strains. This is crucial because it is a proof of concept, showing that the oxidative reactions caused by the ozone enables new antibiotic molecules to function against bacteria that previously were essentially immune to their effects. By increasing the number of effective antibiotics and potentially circumventing developed resistances, this combination approach can provide means to repurpose antibiotics that were no longer effective.

Finally, to ensure that the application of ozone does not have a negative effect on the function of the antibiotics, two tests were conducted. First, Fourier transform infrared (FTIR) readings were taken of both antibiotic samples before and after 8 h of ozone exposure and showed no change in molecular structure. Second, antibacterial tests using vancomycin and linezolid on G + ve bacteria before and after 8 h of ozone exposure also show no change in efficacy due to prolonged ozone exposure at 4 mg/h. These two tests further validate that the gaseous ozone delivered through the patch did not cause adverse effects to the antibiotic compounds. See supporting information for more details.

### Biocompatibility of adjunct therapies

It was previously shown that long term (6 h) exposure of human fibroblast cells to gaseous ozone produced at 4 mg/h showed no signs of stress or reduced viability in the cells. Here, was also important to assess the safety of the combination therapy of topical antibiotics as gaseous ozone at these levels and validate that they combination therapy does not create any cytotoxic compounds. By studying the biocompatibility, we were able to show that the treatment system is both effective and safe to use. To validate this, a systemic biocompatibility tests on human fibroblast cells was conducted, in which cells were either exposed to the ozone therapy (with or without antibiotics) or left as a control. In each case, cell samples were exposed to test inhibitory concentration of solution containing either linezolid, vancomycin or no antibiotic. Tests were conducted under the same 6 h and 4 mg/h ozone parameters as the antibacterial studies. Figure 7c shows the results of an in vitro MTT assay and live/dead staining (Fig. 7d) that were performed to evaluate the viability of fibroblast cells (normalized against the control group) treated with antibiotics in combination with antibiotic eluting electro-spun nanofibers. The viability percentage on cells treated with ozone, ozone + vancomycin, ozone + linezolid, was 98.6%, 99%, and 98.2% respectively after 1 day and 98.8%, 98.5%, and 97.2% respectively after 7 days. These results show that the combination treatment approach did not have any adverse effects on the viability of fibroblast cells.

## Conclusions

In this work, we present for the first time a portable device for integrated application of adjunct ozone and antibiotic therapy targeting resistant strains of G − ve bacteria. The novel combined delivery system for both antibiotic and gaseous ozone is achieved through use of fast-dissolving PVA nanofibers containing antibiotics. The development, structure, and permeability of the NF mat was seen to be very conducive to the adjunct treatment. Optimization of ozone levels applied by the system was performed to achieve treatment with high antibacterial efficacy with minimal cytotoxicity to human cells. Finally, extensive work was conducted to validate the efficacy of the adjunct therapy between ozone and two antibiotics commonly effective against G+ve bacteria, vancomycin and linezolid. This combination was shown to significantly increase the treatment of the gram-negative bacteria *Pseudomonas aeruginosa* and *E. coli *in vitro*,* while showing no signs of cytotoxicity. This system has great promise to be an effective therapy method for infected dermal wounds and is able to significantly increase the number of treatment options available to clinicians and patients as the prevalence of antibiotic-resistant infections continues to rise. Further systematic in vivo research in this area will provide necessary validation as the technology is developed toward clinical trials and human use. This platform could provide a new opportunity to use ozone combination therapy to make previous antibiotics more effective again. It is a crucial step towards helping resolve the crisis of chronic wounds and treatment of resistant infections.

## Materials and methods

### Patch fabrication

The wound dressing and patch was fabricated using the same process outlined in our previous work^37^. In brief, a mixture of PDMS (Sylgard 184) was diluted 1:5 w/w in heptane and used to coat the rayon-spandex fabric (84.5 mm × 67 mm) that is used to create the dressing to induce hydrophobic properties. After drying, this is bonded to the PDMS backing with the internal dispersion layer used for increasing the ozone-effected area in between. The system was bonded using 3 M 300LSE double sided adhesive strips. The biodegradable drug release NFs were spun from a polyvinyl alcohol (PVA) (selected due to well documented biocompatibility and high solubility in water) and water solution (10% w/w PVA). The antibiotics were added at 1% w/w for both vancomycin hydrochloride (1404-93-9 Chemimpex) and linezolid (165800-03-3 Chemimpex). To generate the NF mesh, the dressing was adhered to the drum of the electrospinning machine (Tong Li Tech TL-Pro-BM). The fibers were spun from a needle using an 18 g tip with 20 kV and − 2 kV potential and 0.65 mL/min flow rate at a tip to collector distance of 14 cm using a 10% w/w PVA in water solution (P1763 fully hydrolyzed PVA or 843,869 partially hydrolyzed PVA (MW = 70,000), Sigma-Aldrich). Fibers were deposited on a substrate fixed to a rotating drum (10 cm diameter, 30 RPM) to reach an antibiotic concentration of 200 µg/cm^2^.

### Nanofiber imaging

Imaging of fibers was conducted to identify structure, size, and pore of the mesh generated. Optical imaging was performed using a Steindorff OM. SEM images were captured with a Hitachi S-4800 Field Emission SEM at 4 kV and 20 mA after samples were sputter-coated with Au–Pd to 36 nm. Image processing allowed for measurement of fiber and pore size in captured images and was done with ImageJ software with the measurement and particle analysis tools.

### Hydrophobicity and porosity

Contact angle measurements used to quantify the sample hydrophobicity were taken in triplicate with a Ramé-Hart Model 290 F1 Advanced Goniometer. Air flow pressure measurements were captured in triplicate with an Omega DPG 4000 pressure sensor and the air flow was generated with a New Era 1000 syringe pump. Constant flow was applied through each sample while pressure was concurrently read to measure the internal pressure build-up due to resistance to flow through the sample.

### Nanofiber dissolution

To measure the dissolution of the PVA NFs, NFs were electrospun as previous describes onto an aluminum foil substrate attached to the Tong Li electrospinning drum. To generate NFs loaded with visible dyes instead of antibiotics for spectrophotometry analysis, dyes were added to PVA at aqueous solubility levels associated with each drug (0.1% Direct Red 80 [Sigma Aldrich 365548) and 0.03% methelyne blue (Sigma Aldrich M9140)] and was spun to obtain a mass of 20 µg/cm^2^. Both dyes were selected to mimic the molecular size of the antibiotics (Supplementary Fig. 1). All other deposition characteristics were kept the same. Samples were cut to size using a PLS6MW laser cutter with a 40 W fiber laser (1.06 μm). To characterize the dissolution rate, 0.3 cm^2^ samples were placed in a 96 well plate and exposed to 300 μL of DI water. One set of samples were removed after 1 min, and each following sample after an additional 2 min. The solution was then shaken to homogenize and 100 μL samples were pipetted into another 96 well plate, and the absorbance was measured with a BMG ClarioSTAR PLUS Spectrophotometer. A similar procedure was replicated for the pH variance experiments, with the DI water being replaced by clear buffer solution (Sigma-Aldrich) with pH values of 6, 7, and 8, and also for studying the effect of degree of hydrolyzation, with P1763 fully hydrolyzed PVA or 843,869 partially hydrolyzed PVA (MW = 70,000). Each sample was measured in triplicate.

Gel dissolution testing, used to mimic wound bed conditions, was conducted using low-melting temperature agarose gel dissolved in water to 0.5% w/w (Sigma-Aldrich). The agarose was then pipetted in 1 mL samples into a 12 well plate and allowed to set. 1 cm^2^ circles of blue (linezolid) NFs on the Al foil substrate were cut to shape using the PLS6MW laser cutter and placed on the gel surface. The first set of samples was removed after 1 min, and then each following sample after an additional 2 min. Gel samples with dissolved fibers were then dissolved by heat exposure and stirred before 100 μL samples were extracted and pipetted into a 96 well plate for optical reading using the BMG ClarioSTAR. Each sample was measured in triplicate.

### Ozone antibacterial and biocompatibility characterizations

The clinical isolates of *P. aeruginosa* (25668) and *E. coli* (25922), two common G − ve pathogens found in SSTIs, were obtained from American Type Culture Collection (ATCC). All media and antibiotics were purchased from Sigma Aldrich (St. Louis, MO). Bacteria cultures were revived from frozen stock in a solution of Tryptic Soy Broth (TSB, Sigma Aldrich, St. Louis, MO) and incubated overnight. A sample from the revived stock was diluted 1:50 in Phosphate Buffered Saline (PBS) to achieve a starting inoculum of approximately 10^7^ CFU/mL. 50 μL samples were pipetted into a 96 well plate such that three new wells could be drawn from at each measurement point. Three such plates were prepared, and one each exposed to ozone therapy at 2 mg/h, 4 mg/h, and 8 mg/h through the previously described gas permeable dressing consisting of the hydrophobic membrane layer, internal dispersion layer, and PDMS backing. Ozone gas was applied continuously at 8 h at room temperature. Each hour, 20 μL samples were drawn from three wells and serially diluted for plating on TSB agar plates.

Biocompatibility experiments were adapted from the procedure previously used by Kasi et al.^66^ The cytocompatibility of ozone treatment was analyzed with NIH/3T3 fibroblast cells (purchased from ATCC) by an enzyme-driven colorimetric assay, CellTiter 96 Aqueous One (Promega). The enzyme uses ATP to drive enzyme function, so only live cells convert the substrate into the compound detectable in the spectrophotometer. The plates were cultured with NIH/3T3 cells in DMEM media with 10% FBS. To test the cytocompatibility, the ozone (2–8 mg/h) is exposed via pads to the cells at day 0. There were 5000 cells/mL suspension on each well. 3 plates were used to study the cell viability for each concentration. In each plate 3 wells were used a replicate. The different ozone concentration was exposed for 6 h inside the cell culture chamber (CO_2_ chamber, 37 °C). The cells without ozone treatment were also used as a control. The DMEM suspension was aspirated from the test wells on each day and covered with 200 µL of MTT reagent. The reagent to media ratio was 20:100 µL for a total of 200 µL to cover the samples. Samples attached with NIH/3T3 cells were allowed to reduce the substrate for 1 h. Then, three aliquots of 100 µL were transferred to a 96 well plate. The optical absorbance of the samples was read at a fixed wavelength of 490 nm in the spectrophotometer, SpectraMax M2 (MolecularDevices, USA), which was calibrated with a blank MTT reagent. Live/Dead imaging was also done to study the cell viability upon the different ozone exposure. The same setup mentioned above in the MTT assay was used to study the visual cell viability. The NIH/3T3 fibroblast cells were grown; the cells were imaged for live/dead using calcein-AM and ethidium homodimer − 1 at different time intervals like day 1, 3 and 7. The NIH/3T3 cells were grown as mentioned above in the MTT analysis, and three identical plates along with control (no treatment) were used for the study. The cells are imaged with the respective filters in Nikon Ti2 Eclipse, equipped with a camera under a 10X optical lens using NIS-Elements D software.

### Adjunct antibacterial and biocompatibility characterizations

To test the antibacterial effects of ozone in combination with antibiotics, the same ATCC 25668 *P. aeruginosa* and ATCC 25922 *E. coli* cultures were inoculated in a solution of Tryptic Soy Broth (TSB, Sigma Aldrich, St. Louis, MO) and incubated overnight. A sample of the mature culture was diluted in new TSB at 1:500 (starting inoculum of approximately 10^6^) to provide nutrients necessary for continued bacterial growth during testing to better mimic the wound environment. The new culture media was then pipetted into test wells in triplicate at 1:10 with either linezolid or vancomycin solution in deionized water at 2000 μg/mL. The final solution in each well had a volume of 50 μL and a concentration of antibiotic of 200 μg/mL.

The bacterial samples were subject to the combination treatment of 4 mg/h ozone with vancomycin at 200 μg/mL and linezolid at 200 μg/mL. This concentration of antibiotic was selected to provide a high (10×) concentration to enable sufficient antibiotic strength for treatment of normally resistant strains of bacteria^41,67–70^. During testing, the test samples were kept at 37 °C to ensure proper bacteria growth was possible. At 2, 4, and 6 h, 20 μL samples were withdrawn from the designated set of wells and plated on TSB agar plates. Bacterial colonies were counted after being incubated overnight at 37 °C. Experimental adjunct therapy samples were compared against treatment of only ozone, and control cultures exposed to antibiotics without the ozone.

Adjunct therapy biocompatibility experiments followed the procedure outlined above for ozone therapy^66^. In short, ozone adjunct treatment was analyzed with NIH/3T3 fibroblast cells purchased from ATCC using the same colorimetric assay. Cultured plates containing cells at 5000 cells/mL were tested with 4 mg/h ozone and 200 μg/mL vancomycin or 200 μg/mL linezolid on day 0 and compared to cells without any treatment. Combination treatment was exposed for 6 h inside the cell culture chamber at 37 °C. MTT assay was added at 20:100 µL in each sample and allowed to reduce the substrate for 1 h. Three 100 µL samples were transferred to a 96 well plate for optical absorbance measurements at 490 nm using the spectrophotometer. Additionally, samples were exposed to Live/Dead imaging after 1, 3, and 7 days and compared to control results. Staining images were taken using a 10× optical lens.

### Statistical analysis

A one-way ANOVA tests was used to determine statistical significance of the samples (α = 0.05) for antibacterial tests. Dunnet’s Test for multiple comparison was performed afterward to verify statistical difference between different conditions and time points.

## Supplementary Information

Supplementary Information.

## Data Availability

The datasets generated during and/or analyzed during the current study are available from the corresponding author on reasonable request.
