# Supplementary material for: Wearable adjunct ozone and antibiotic therapy system for treatment of Gram-negative dermal bacterial infection
Source: Sci Rep. 2022 Aug 17;12:13927. doi: 10.1038/s41598-022-17495-3 (PMC9385669; doi:10.1038/s41598-022-17495-3)
Supplement: Supplementary file 1 — Supplementary Information. [file 41598_2022_17495_MOESM1_ESM.pdf]

Supplementary Information for:

Wearable Adjunct Ozone and Antibiotic Therapy System for Treatment of Gram-negative Dermal Bacterial Infection

Authors: Alexander Roth, Murali Kannan Maruthamuthu, Sina Nejati, Akshay Krishnakumar, Vidhya Selvamani, Sotoudeh Sedaghat, Juliane Nguyen, Mohamed N. Seleem, Rahim Rahimi

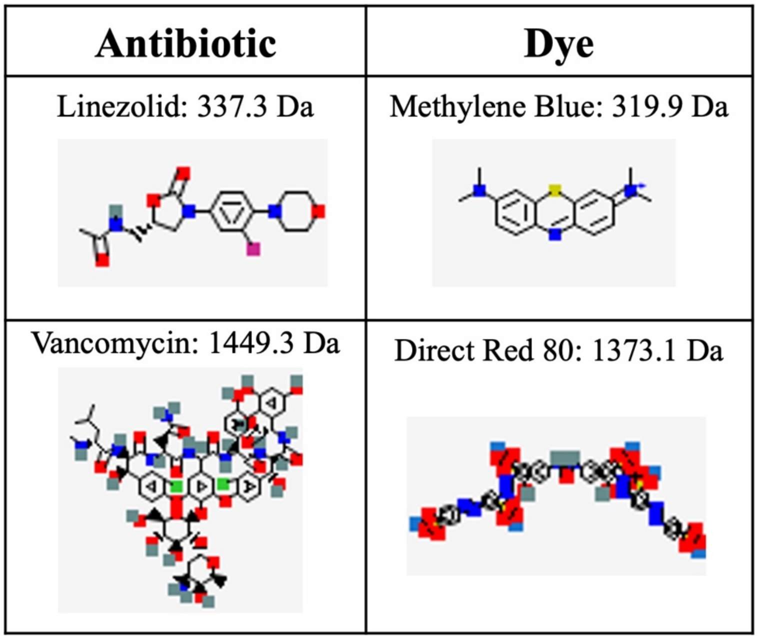

Supplementary Figure S1. Comparison of molecular weight and shape of antibiotic molecules with dye chosen to simulate in dissolution[1–4].

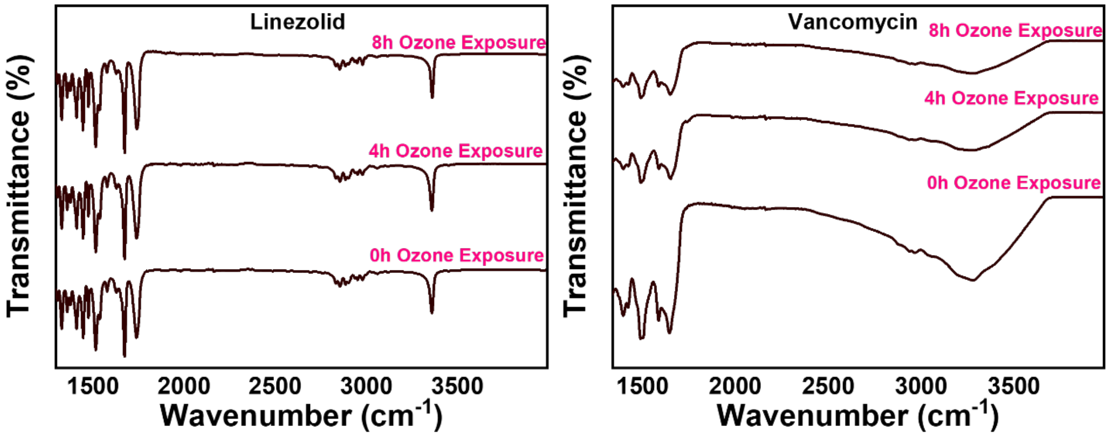

Supplementary Figure S2. Fourier Transform Infrared spectra of Vancomycin and Linezolid with 0, 4, and 8 hours of ozone exposure.

FTIR spectra of the linezolid and vancomycin antibiotics with the ozone exposure time of 0, 4 and 8 h is shown in Fig. Both FTIR spectra showed a strong signal between 3050 to 3550 cm^{-1}, which may be attributed to the alcohol of PVA chain. Similarly, the peak observed around 2920 cm^{-1} could probably be due to the R-CH_2 stretching of the PVA chains.
On the other hand, the weak band stretching at 1675 cm^{-1} and 1750 cm^{-1} in fig (a) could be accredited to the C=O stretching of the acetamide carbon and oxazolidinone carbonyl of linezolid respectively [5,6]. Identical peaks were observed by Shah *et al.*[7]. Similarly, the band stretching at 1650 cm^{-1} and 1512 cm^{-1} could be due to the C=O and C-N stretching of vancomycin [8].

No significant change in the FTIR spectra was observed was observed elucidating the stability of the antibiotics under ozonized environment.

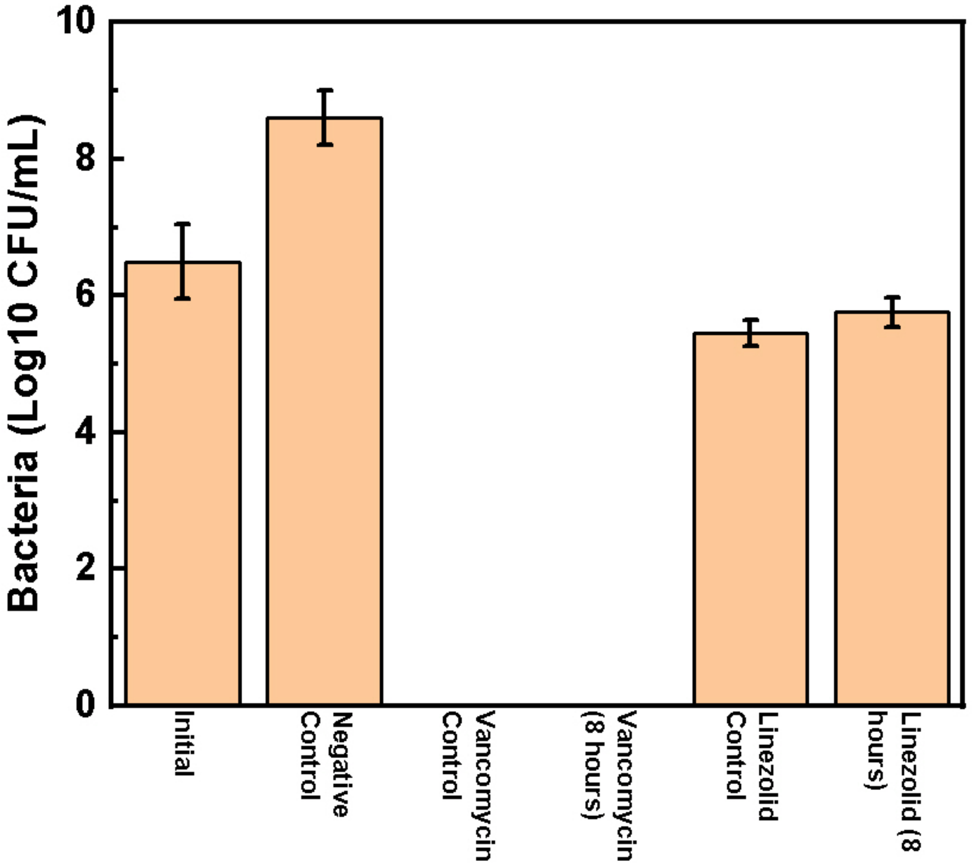

Supplementary Figure S3. Antibacterial response of Vancomycin and Linezolid before and after 8 hours of ozone exposure. Error bars denote standard deviation.

The effect of extended exposure of Vancomycin and Linezolid to ozone in solution was investigated. To do this, a stock solution of both antibiotics was dissolved in deionized (DI) water at 200µg/mL. 2.5mL of stock solution was added to 2 wells of a 6 well plate for each antibiotic. These wells were exposed to ozone gas generated at 4mg/hr for 8 hours. After 8 hours, samples from each condition were collected and stored.

The antibacterial properties of Vancomycin and Linezolid were tested as follows. An overnight culture of *S. aureus* (ATCC 25923) was revived from frozen stock in Tryptic Soy Broth (TSB) purchased from Sigma Aldrich (St. Louis, MO). Sample from the revived culture was diluted 1:1000 in fresh TSB and added at 90µL to a 96 well plate. 10µL of the corresponding antibiotic stock solution was added to each well such that each condition was tested in triplicate. The 96 well plate containing the samples was then incubated at 37°C for 24 hours. Samples were collected from each well and serially diluted for plating and counting on TSB agar plates.

Results indicate that extended exposure to ozone caused no significant alteration to the antimicrobial performance of either antibiotic. The difference in antimicrobial performance between the two antibiotics is merely due to their classification. Linezolid is classified as a

bacteriostatic compound against *S. aureus*, which means its action prevents the replication of bacteria, but without an outside effect to induce cell death (i.e. human immune system), the number of bacteria won't decrease significantly(52). Vancomycin, on the other hand, is classified as a bactericidal compound against *S. aureus* which leads to cell death, as seen in the test(53).

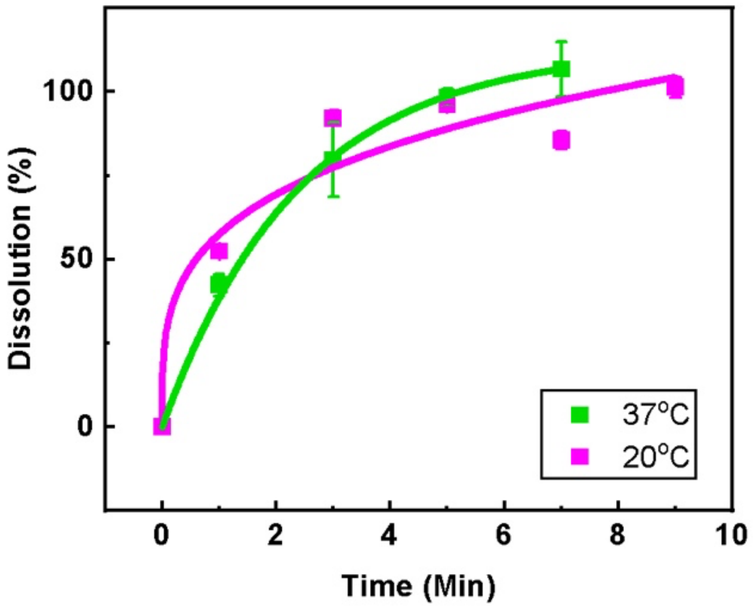

Supplementary Figure S4. Dissolution characterization of PVA NFs at 20°C and 37°C using NFs containing methylene blue dye. Error bars denote standard deviation.

The effect of temperature on the dissolution of PVA NFs was characterized. This was to understand how elevated wound bed temperatures (commonly between 30.2-33.0°C) would affect the dissolution of the PVA and release of the antibiotics into the system[9]. It is known that in general, increasing temperature will cause PVA to dissolve faster. As shown by *Finch*, partially hydrolyzed PVA, such as that used in this study, already has a near complete solubility in the range of 20-37°C and shows minimal increase[10]. As such, the data here supports a minimal increase in the dissolution rate due to increased temperature, though any increase due to this will serve to enable a faster dissolution of fibers which is desirable for this application.

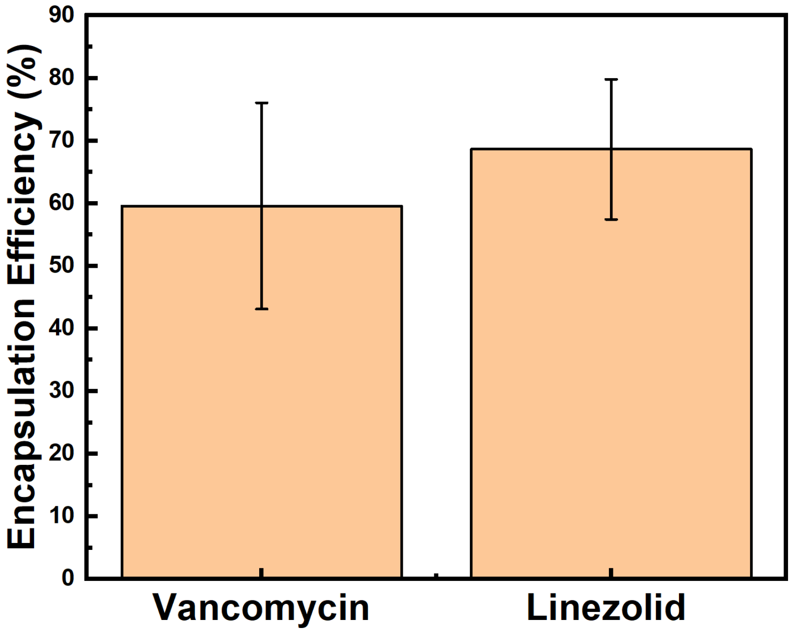

Supplementary Figure S5. Encapsulation efficiency of antibiotics in PVA nanofibers. Error bars denote standard deviation.

Encapsulation efficiency was measured by comparing stock solutions of both antibiotics at 200 µg/mL in DI water to test solutions prepared by dissolving the electrospun drug-eluting NFs in DI water such that 1 cm^2 of NFs were dissolved in 1 mL for a final target concentration of 200 µg/mL. The amount of antibiotic in both stock solutions and test solutions was measured using UV spectroscopy (250nm for linezolid and 281nm for vancomycin) [1,11]. To account for the background absorption due to the PVA NFs, 1cm^2 of PVA NFs without any additive was added to the stock antibiotic solution and a baseline solution of DI water with equivalent concentration of PVA NFs was used to compensate for the background in the experimental readings. The absorption values of the stock and test solutions were measured in triplicate using a NanoDrop 1000. 2µL samples were analyzed with the Nanodrop between 220-400nm and the efficiency was calculated by

$$A_{test}/A_{stock} * 100 \quad (1)$$

where A_{test} and A_{stock} are the absorbance values of the test and stock solutions respectively measured at the peak absorption wavelengths for vancomycin and linezolid listed above. The results are included in **Fig. S5** shown above, with vancomycin exhibiting an encapsulation efficiency of 59.6% and linezolid 68.6%. These encapsulation efficiency values are similar to those found in literature for similar structures[12–17].

References

1. PubChem Compound Summary for CID 441401, Linezolid. *National Center for Biotechnology Information* https://pubchem.ncbi.nlm.nih.gov/compound/Linezolid.
2. PubChem Summary for CID 14969, Vancomycin. *National Center for Biotechnology Information* https://pubchem.ncbi.nlm.nih.gov/compound/Vancomycin.
3. PubChem Compound Summary for CID 75783, Direct Red 80. *National Center for Biotechnology Information* https://pubchem.ncbi.nlm.nih.gov/compound/Direct-Red-80.
4. PubChem Compound Summary for CID 6099, Methylene blue. *National Center for Biotechnology Information* https://pubchem.ncbi.nlm.nih.gov/compound/Methylene-blue.
5. Frelek, J. *et al.* Distinguishing between polymorphic forms of linezolid by solid-phase electronic and vibrational circular dichroism. *Chem. Commun.* **48**, 5295–5297 (2012).
6. Nuñez, Y. A. R. *et al.* Preparation of hydrogel/silver nanohybrids mediated by tunable-size silver nanoparticles for potential antibacterial applications. *Polymers (Basel).* **11**, (2019).
7. Shah, S., Maheshwari, H., Soniwala, M. & Chavda, J. Pulmonary Delivery of Linezolid Nanoparticles for Treatment of Tuberculosis: Design, Development, and Optimization. *J. Pharm. Innov.* (2020) doi:10.1007/s12247-020-09491-9.
8. Mohamed, H. B., El-Shanawany, S. M., Hamad, M. A. & Elsabahy, M. Niosomes: A Strategy toward Prevention of Clinically Significant Drug Incompatibilities. *Sci. Rep.* **7**, 1–14 (2017).
9. Gethin, G. *et al.* What is the 'normal' wound bed temperature? A scoping review and new hypothesis. *Wound Repair Regen.* **29**, 843–847 (2021).
10. Finch, C. A. SOME PROPERTIES OF POLYVINYL ALCOHOL AND THEIR POSSIBLE APPLICATIONS. *Chem Technol Water-Soluble Polym* 287–306 (1983) doi:10.1007/978-1-4757-9661-2_17/COVER/.
11. Pande, S. & Parikh, J. R. Development and Validation of UV- Spectrophotometric Method for estimation of Vancomycin Hydrochloride. *J. Drug Deliv. Ther.* **9**, 116–118 (2019).
12. Dorati, R. *et al.* Tubular Electrospun Vancomycin-Loaded Vascular Grafts: Formulation Study and Physicochemical Characterization. *Polym. 2021, Vol. 13, Page 2073* **13**, 2073 (2021).
13. Mohamady Hussein, M. A. *et al.* Dual-drug delivery of Ag-chitosan nanoparticles and phenytoin via core-shell PVA/PCL electrospun nanofibers. *Carbohydr. Polym.* **270**, 118373 (2021).
14. Eren Boncu, T. *et al.* In vitro and in vivo evaluation of linezolid loaded electrospun PLGA and PLGA/PCL fiber mats for prophylaxis and treatment of MRSA induced prosthetic infections. *Int. J. Pharm.* **573**, 118758 (2020).
15. Eren Boncu, T., Ozdemir, N. & Uskudar Guclu, A. Electrospinning of linezolid loaded PLGA nanofibers: effect of solvents on its spinnability, drug delivery, mechanical properties, and antibacterial activities. *Drug Dev. Ind. Pharm.* **46**, 109–121 (2020).
16. Razzaq, A. *et al.* Development of Cephradine-Loaded Gelatin/Polyvinyl Alcohol Electrospun Nanofibers for Effective Diabetic Wound Healing: In-Vitro and In-Vivo Assessments. *Pharm. 2021, Vol. 13, Page 349* **13**, 349 (2021).
17. Vashisth, P. *et al.* Ofloxacin loaded gellan/PVA nanofibers - Synthesis, characterization

and evaluation of their gastroretentive/mucoadhesive drug delivery potential. *Mater. Sci. Eng. C* **71**, 611–619 (2017).
